# Supplementary material for: Hybrid Models and Biological Model Reduction with PyDSTool
Source: PLoS Comput Biol. 2012 Aug 9;8(8):e1002628. doi: 10.1371/journal.pcbi.1002628 (PMC3415397; doi:10.1371/journal.pcbi.1002628)
Supplement: Text S4 — Complete source code for the PyDSTool package (version 0.88.120504). Includes API documentation and help files linking to web pages. This file is identical to the current public release on Sourceforge.net. (ZIP) [file pcbi.1002628.s004.zip › PyDSTool/html/PyDSTool.MProject-pysrc.html]

xml version="1.0" encoding="ascii"?


PyDSTool.MProject


| Home | Trees | Indices | Help | PyDSTool |
| --- | --- | --- | --- | --- |

Package PyDSTool ::
Module MProject

[hide private]

[frames] | no frames]

# Source Code for Module PyDSTool.MProject

```
   1  """Modelling project and associated classes. 
   2   
   3     For aiding model estimation using (hybrid) dynamical systems. 
   4   
   5     Robert Clewley, September 2007. 
   6  """ 
   7   
   8  import copy 
   9  import sys, traceback 
  10  import numpy as npy 
  11   
  12  # PyDSTool imports 
  13  import Model, Generator, Events, ModelSpec, ModelConstructor, Symbolic, \ 
  14         Trajectory 
  15  import utils, common, parseUtils 
  16  from errors import * 
  17   
  18  # -------------------- 
  19   
  20  # public exports 
  21   
  22  _classes = ['GenTransform', 'ModelTransform', 'ModelManager', 'feature', 
  23              'ql_feature_node', 'ql_feature_leaf', 'qt_feature_node', 
  24              'qt_feature_leaf', 'binary_feature', 'always_feature', 
  25              'condition', 'context', 'ModelLibrary', 'GeneratorInterface', 
  26              'ModelInterface', 'extModelInterface', 'intModelInterface'] 
  27   
  28  _functions = ['extract_from_model'] 
  29   
  30  __all__ = _classes + _functions 
  31   
  32  # --------------------------------------------------------------------- 
  33   
  34  # do we need this? 


35 -def extract_from_model(model):


36      try: 
  37          r = copy.copy(model._mspecdict) 
  38      except AttributeError: 
  39          raise ValueError("Incompatible Model argument -- it contains " 
  40                           "no ModelSpec info") 
  41      for key in ['algparams', 'mspecdict', 'targetlang']: 
  42          assert key in r 
  43      # add inputs information to r, i.c.'s and text description 
  44      return r

  45   
  46   
  47   
  48  # ---------------------------------------------------------------------------- 
  49   


50 -class ModelLibrary(object):


51      """Store a set of related candidate model types, and within each, represent 
  52      various relevant "dimensions" along which the model can be augmented 
  53      structurally.""" 


54 -    def __init__(self, name, spectype, indepdomain, depdomain, 
  55                   pars=None, description=''):


56          self.name = name 
  57          self.spectype = spectype 
  58          # instances is name -> spec mapping 
  59          self.instances = {} 
  60          self.indepdomain = indepdomain 
  61          self.depdomain = depdomain 
  62          self.pars = pars 
  63          self.description = ''

  64   


65 -    def __getitem__(self, name):


66          return self.instances[name]

  67   


68 -    def add_spec(name, specs):


69          if not isinstance(specs, common._seq_types): 
  70              specs = [specs] 
  71          for spec in specs: 
  72              if isinstance(spec, self.spectype): 
  73                  self.instances[spec.name] = spec 
  74                  spec.library_tag = self.name 
  75              else: 
  76                  raise PyDSTool_TypeError("Spec of wrong type")

  77   


78 -    def __str__(self):


79          return "Model Library %s: %s"%(self.name, self.description)

  80   
  81   


82 -class GenTransform(object):


83      """Generator Transformer class. 
  84      Acts on GDescriptor objects that define Generators. 
  85      For these, the only non-trivial transformations are inside the modelspec 
  86      attribute. 
  87      """ 


88 -    def __init__(self, name, gen, model_icvalues=None, model_parvalues=None, 
  89                   model_inputs=None):


90          if not isinstance(gen, ModelConstructor.GDescriptor): 
  91              raise TypeError("GenTransform must be initialized with a " 
  92                              "GDescriptor object") 
  93          self.orig_gen_name = name 
  94          self.orig_gen = gen 
  95          self.trans_gen = copy.deepcopy(gen) 
  96          self.changelog = [] 
  97          if model_icvalues is None: 
  98              self.model_icvalues = {} 
  99          else: 
 100              self.model_icvalues = model_icvalues 
 101          if model_parvalues is None: 
 102              self.model_parvalues = {} 
 103          else: 
 104              self.model_parvalues = model_parvalues 
 105          if model_inputs is None: 
 106              self.model_inputs = {} 
 107          else: 
 108              self.model_inputs = model_inputs

 109   


110 -    def remove(self, obj):


111          """Remove component, parameter, variable, input, function""" 
 112          self.trans_gen.modelspec.remove(obj) 
 113          self.changelog.append(common.args(action='remove', target=obj.name))

 114   


115 -    def add(self, parent_name, obj):


116          """Add component, parameter, variable, input, function""" 
 117          # resolve parent_name structure 
 118          self.trans_gen.modelspec.add(obj, parent_name) 
 119          self.changelog.append(common.args(action='add', target=obj.name))

 120   


121 -    def findStaticVars(self):


122          """Find RHSfuncSpec variables with RHS=0""" 
 123          return [v for v in self.trans_gen.modelspec.search(Var) if \ 
 124                  gen.modelspec[v].spec.specStr == '0']

 125   


126 -    def changeTargetGen(self, target):


127          """Change target generator type. Target is a string name of the Generator 
 128          class.""" 
 129          self.trans_gen.target = target

 130   


131 -    def changeDomain(self, obj_name, domain):


132          """Change valid domain of a quantity""" 
 133          try: 
 134              self.trans_gen.modelspec[obj_name].setDomain(domain) 
 135          except (KeyError, AttributeError): 
 136              raise PyDSTool_TypeError("Invalid quantity for domain change") 
 137          self.changelog.append(common.args(action='changeDomain', \ 
 138                                            target=obj_name, pars=(domain,)))

 139   


140 -    def redefineQuantity(self, obj_name, specstr):


141          """Redefine a Quantity using a new specification string, 
 142          leaving its type unchanged. 
 143          """ 
 144          try: 
 145              obj = self.trans_gen.modelspec[obj_name] 
 146          except KeyError: 
 147              raise PyDSTool_ValueError("Unknown object") 
 148          try: 
 149              obj.spec.redefine(specstr) 
 150          except AttributeError: 
 151              raise PyDSTool_TypeError("Invalid quantity for redefinition") 
 152          self.trans_gen.modelspec.remove(obj_name) 
 153          if parseUtils.isHierarchicalName(obj_name): 
 154              parts = obj_name.split(parseUtils.NAMESEP) 
 155              parent_name = ".".join(parts[:-1]) 
 156              obj.rename(".".join(parts[1:])) 
 157          else: 
 158              parent_name = None 
 159          self.trans_gen.modelspec.add(obj, parent_name) 
 160          self.changelog.append(common.args(action='redefineQuantity', \ 
 161                                            target=obj_name, pars=(specstr,)))

 162   


163 -    def convertQuantity(self, obj_name, targetType, targetSpecType=None):


164          """Convert quantity between parameter, variable, or input types. 
 165          If parameter -> variable, the RHS will be set to zero ('static' 
 166          variable). 
 167          """ 
 168          try: 
 169              obj = self.trans_gen.modelspec[obj_name] 
 170          except KeyError: 
 171              raise PyDSTool_ValueError("Unknown object") 
 172          if parseUtils.isHierarchicalName(obj_name): 
 173              parent_name = obj_name.split(parseUtils.NAMESEP)[0] 
 174          else: 
 175              parent_name = '' 
 176          try: 
 177              currentType = obj.typestr 
 178              assert currentType in ('par', 'var', 'input') 
 179              assert targetType in ('par', 'var', 'input') 
 180          except (AttributeError, AssertionError): 
 181              raise PyDSTool_TypeError("Only convert between parameter, variable or " 
 182                              "input quantity types") 
 183          if targetType == currentType: 
 184              if currentType != 'var' or obj.specType is None: 
 185                  # either (1) par->par, (2) input->input, or 
 186                  # (3) var->var with no specType to change 
 187                  # In any of these cases, nothing to do 
 188                  return 
 189          if currentType == 'var': 
 190              assert obj.specType in ('RHSfuncSpec', 'ExpFuncSpec'), \ 
 191                     "Cannot process implicit function specs" 
 192          if targetType == 'var': 
 193              assert targetSpecType in ('RHSfuncSpec', 'ExpFuncSpec'), \ 
 194                     "target specType must be RHSfuncSpec of ExpFuncSpec only" 
 195          if targetType == 'par': 
 196              if currentType == 'var' and obj_name in self.model_icvalues: 
 197                  # use existing initial condition for variable as parameter value 
 198                  new_obj = Symbolic.Par(repr(self.model_icvalues[obj_name]), 
 199                                         obj.name, domain=obj.domain) 
 200                  #del(self.trans_gen.icvalues[obj_name]) 
 201              else: 
 202                  #if currentType == 'input' and name in self.model_inputs: 
 203                  #    del(self.model_inputs[obj_name]) 
 204                  new_obj = Symbolic.Par(obj.name, domain=obj.domain) 
 205          elif targetType == 'input': 
 206              #if currentType == 'var' and name in self.model_icvalues: 
 207              #    del(self.model_icvalues[name]) 
 208              #elif currentType == 'par' and name in self.model_parvalues: 
 209              #    del(self.model_parvalues[name]) 
 210              new_obj = Symbolic.Input(obj.name, domain=obj.domain) 
 211          elif targetType == 'var': 
 212              new_obj = Symbolic.Var('0', obj_name, domain=obj.domain, 
 213                                     specType=targetSpecType) 
 214              if currentType == 'par': 
 215                  try: 
 216                      val = float(obj.spec()) 
 217                  except ValueError: 
 218                      if obj_name in self.model_parvalues: 
 219                          val = self.model_parvalues[obj_name] 
 220                      else: 
 221                          val = None 
 222                  if val is not None: 
 223                      # par had a value already, so use that for the 
 224                      # initial condition of this var 
 225                      self.model_icvalues[obj_name] = val 
 226              #elif currentType == 'input' and name in self.model_inputs: 
 227              #    del(self.model_inputs[obj_name]) 
 228          else: 
 229              raise PyDSTool_TypeError("Invalid conversion") 
 230          self.trans_gen.modelspec.remove(obj_name) 
 231          self.trans_gen.modelspec.add(new_obj, parent_name) 
 232          self.changelog.append(common.args(action='convertQuantity', 
 233                                  target=obj_name, 
 234                                  pars=(targetType, targetSpecType)))

 235   


236 -    def convertComponent(self, obj_name, targetType):


237          """Convert component object to given type (provide actual type), 
 238          provided the new type is compatible with the old one. 
 239          """ 
 240          try: 
 241              obj = self.trans_gen.modelspec[obj_name] 
 242          except KeyError: 
 243              raise PyDSTool_ValueError("Unknown object") 
 244          if parseUtils.isHierarchicalName(obj_name): 
 245              parent_name = obj_name.split(parseUtils.NAMESEP)[0] 
 246          else: 
 247              parent_name = '' 
 248          currentType = common.className(obj) 
 249          if not isinstance(obj, ModelSpec.ModelSpec): 
 250              raise PyDSTool_TypeError("Only convert ModelSpec Component objects") 
 251          if targetType == currentType: 
 252              # nothing to do 
 253              return 
 254          if not obj.compatibleContainers == targetType.compatibleContainers or \ 
 255             not obj.compatibleSubcomponents == targetType.compatibleSubcomponents: 
 256              raise PyDSTool_TypeError("Only convert to equivalently-compatible type") 
 257          new_obj = targetType(obj.name) 
 258          new_obj.__dict__.update(obj.__dict__) 
 259          self.trans_gen.modelspec.remove(obj) 
 260          self.trans_gen.modelspec.add(new_obj, parent_name) 
 261          self.changelog.append(common.args(action='convertComponent', target=obj.name, 
 262                                     pars=(common.className(targetType),)))

 263   
 264   


265 -    def makeStaticVar(self, obj_name):


266          """Force RHSfuncSpec variable to have RHS=0. 
 267          """ 
 268          try: 
 269              obj = self.trans_gen.modelspec[obj_name] 
 270          except KeyError: 
 271              raise PyDSTool_ValueError("Unknown object") 
 272          if parseUtils.isHierarchicalName(obj_name): 
 273              parent_name = obj_name.split(parseUtils.NAMESEP)[0] 
 274          else: 
 275              parent_name = '' 
 276          if obj.typestr != 'var' and obj.specType != 'RHSfuncSpec': 
 277              raise PyDSTool_TypeError("Invalid variable object passed") 
 278          new_obj = Symbolic.Var('0', obj.name, domain=obj.domain, 
 279                                 specType='RHSfuncSpec') 
 280          self.trans_gen.modelspec.remove(obj) 
 281          self.trans_gen.modelspec.add(new_obj, parent_name) 
 282          self.changelog.append(common.args(action='makeStaticVar', 
 283                                            target=obj.name))

 284   


285 -    def unresolved(self):


286          """Returns the generator spec's remaining free symbols. 
 287          """ 
 288          return self.trans_gen.validate()[1]

 289   


290 -    def commit(self, new_name):


291          """Verifies completeness of definition before returning new 
 292          generator spec. 
 293          """ 
 294          if self.changelog == []: 
 295              raise PyDSTool_ValueError("No changes made") 
 296          validated, freeSymbols = self.trans_gen.validate() 
 297          if validated: 
 298              self.trans_gen.modelspec.name = new_name 
 299              self.trans_gen.orig_name = self.orig_gen.modelspec.name 
 300              self.trans_gen.changelog = copy.copy(self.changelog) 
 301              return self.trans_gen 
 302          else: 
 303              print "Remaining free symbols: ", freeSymbols 
 304              raise PyDSTool_ValueError("New Generator spec cannot be committed")

 305   
 306   
 307   


308 -class ModelTransform(object):


309      """Model Transformer class. 
 310      """ 


311 -    def __init__(self, name, model):


312          if not isinstance(model, ModelConstructor.MDescriptor): 
 313              raise TypeError("ModelTransform must be initialized with a " 
 314                              "MDescriptor object") 
 315          self.orig_model_name = name 
 316          self.orig_model = model 
 317          self.trans_model = copy.deepcopy(model) 
 318          self.changelog = [] 
 319          self.gentrans = None   # transactions for any GenTransforms

 320   


321 -    def remove(self, obj):


322          "Remove hybrid model generator" 
 323          self.trans_model.remove(obj) 
 324          self.changelog.append(common.args(action='remove', 
 325                                            target=obj.modelspec.name))

 326   


327 -    def add(self, obj):


328          "Add hybrid model generator" 
 329          self.trans_model.add(obj) 
 330          self.changelog.append(common.args(action='add', 
 331                                            target=obj.modelspec.name))

 332   


333 -    def open_gentrans(self, name):


334          """Open a generator transformation transaction""" 
 335          if self.gentrans is None: 
 336              if name in self.trans_model.generatorspecs: 
 337                  self.gentrans = GenTransform(name, 
 338                                 self.trans_model.generatorspecs[name], 
 339                                 self.trans_model.icvalues, 
 340                                 self.trans_model.parvalues, 
 341                                 self.trans_model.inputs) 
 342              else: 
 343                  raise KeyError('Generator %s does not exist in registry'%name) 
 344              return self.trans_model.generatorspecs[name] 
 345          else: 
 346              raise AssertionError("A transaction is already open")

 347   


348 -    def rollback_gentrans(self):


349          if self.gentrans is None: 
 350              raise AssertionError("No transaction open") 
 351          else: 
 352              self.gentrans = None

 353   


354 -    def commit_gentrans(self, new_name, description=''):


355          if self.gentrans is None: 
 356              raise AssertionError("No transaction open") 
 357          else: 
 358              self.add(self.gentrans.commit(new_name)) 
 359              del self.trans_model.generatorspecs[self.gentrans.orig_gen_name] 
 360              # update these if they were changed by gen transformation 
 361              self.trans_model.icvalues = self.gentrans.model_icvalues 
 362              self.trans_model.parvalues = self.gentrans.model_parvalues 
 363              self.trans_model.inputs = self.gentrans.model_inputs 
 364              self.gentrans = None

 365   


366 -    def unresolved(self):


367          """Returns the unresolved inconsistencies in model's internal 
 368          interfaces. 
 369          """ 
 370          return self.trans_model.validate()[1]

 371   


372 -    def commit(self, new_name):


373          """Verifies internal interface consistency before returning new 
 374          model spec. 
 375          """ 
 376          if self.changelog == []: 
 377              raise PyDSTool_ValueError("No changes made") 
 378          validated, inconsistencies = self.trans_model.validate() 
 379          if validated: 
 380              self.trans_model.name = new_name 
 381              self.trans_model.orig_name = self.orig_model.name 
 382              self.trans_model.changelog = copy.copy(self.changelog) 
 383              return self.trans_model 
 384          else: 
 385              print "Internal interface inconsistencies: ", inconsistencies 
 386              raise PyDSTool_ValueError("New Model spec cannot be committed")

 387   
 388   
 389  # ---------------------------------------------------------------------------- 
 390   


391 -class ModelManager(object):


392      """Model management and repository class.""" 
 393   


394 -    def __init__(self, name):


395          assert isinstance(name, str) 
 396          self.proj_name = name 
 397          # registry of model descriptors and instances that form the project 
 398          self._mReg = MReg() 
 399          # transformation transaction holder 
 400          self.trans = None 
 401          # shortcut to model instances 
 402          self.instances = {}

 403   


404 -    def get_desc(self, name):


405          if name in self._mReg: 
 406              return self._mReg.descs[name] 
 407          else: 
 408              raise KeyError('Model %s does not exist in registry'%name)

 409   


410 -    def __getitem__(self, name):


411          if name in self._mReg: 
 412              return self._mReg[name] 
 413          else: 
 414              raise KeyError('Model %s does not exist in registry'%name)

 415   


416 -    def add(self, model_desc):


417          if not isinstance(model_desc, ModelConstructor.MDescriptor): 
 418              raise TypeError("Invalid model descriptor") 
 419          if not model_desc.validate(): 
 420              raise ValueError("Model definition not successfully validated") 
 421          if model_desc not in self._mReg: 
 422              self._mReg.add(model_desc) 
 423          else: 
 424              raise KeyError('Model with this name already exists in registry')

 425   


426 -    def remove(self, name):


427          if name in self._mReg: 
 428              del(self._mReg[name]) 
 429          else: 
 430              raise KeyError('Model with this name does not exist in registry')

 431   
 432      __delitem__ = remove 
 433   


434 -    def open_trans(self, name):


435          """Open a model transformation transaction""" 
 436          if self.trans is None: 
 437              self.trans = ModelTransform(name, self.__getitem__(name)) 
 438              return self._mReg.descs[name] 
 439          else: 
 440              raise AssertionError("A transaction is already open")

 441   


442 -    def rollback_trans(self):


443          if self.trans is None: 
 444              raise AssertionError("No transaction open") 
 445          else: 
 446              self.trans = None

 447   


448 -    def commit_trans(self, new_name, description=''):


449          if self.trans is None: 
 450              raise AssertionError("No transaction open") 
 451          else: 
 452              self.add(self.trans.commit(new_name)) 
 453              self.trans = None

 454   


455 -    def build(self, name, icvalues=None, parvalues=None, 
 456                inputs=None, tdata=None):


457          try: 
 458              mdesc = copy.deepcopy(self._mReg[name]) 
 459          except KeyError: 
 460              raise KeyError("No such model description") 
 461          for gd in mdesc.generatorspecs.values(): 
 462              gd.modelspec.flattenSpec(ignoreInputs=True, force=True) 
 463          filt_keys = ('userevents', 'userfns', 'unravelInfo', 
 464                   'inputs', 'checklevel', 'activateAllBounds', 
 465                   'generatorspecs', 'indepvar', 
 466                   'parvalues', 'icvalues', 'reuseTerms', 
 467                   'withJac', 'withJacP', 'tdata', 
 468                   'abseps', 'eventtol', 'eventPars', 
 469                   'withStdEvts', 'stdEvtArgs') 
 470          if icvalues is not None: 
 471              mdesc.icvalues.update(icvalues) 
 472          if parvalues is not None: 
 473              mdesc.parvalues.update(parvalues) 
 474          if inputs is not None: 
 475              mdesc.inputs.update(inputs) 
 476          if tdata is not None: 
 477              mdesc.tdata = tdata 
 478          if not mdesc.isinstantiable(True): 
 479              raise ValueError("Model description incomplete: not instantiable") 
 480          ## would like ModelConstructor to be able to deal with the remaining 
 481          # keys of mdesc so that all the information in mdesc gets passed into 
 482          # the _mspec attribute of the instantiated model, otherwise mdesc needs 
 483          # to be stored somewhere else. 
 484          mc = ModelConstructor.ModelConstructor(mdesc.name, 
 485                                  **common.filteredDict(dict(mdesc), filt_keys)) 
 486          assert len(mdesc.generatorspecs) > 0, "No Generator descriptions found" 
 487          for gdname, gd in mdesc.generatorspecs.iteritems(): 
 488              if gd.userEvents is not None: 
 489                  mc.addEvents(gdname, gd.userEvents) 
 490              if gd.userFunctions is not None: 
 491                  mc.addFunctions(gdname, gd.userFunctions) 
 492              if gd.userEventMaps is not None: 
 493                  for em in gd.userEventMaps: 
 494                      try: 
 495                          # in case evmap included 
 496                          evname, target, evmap = em 
 497                      except ValueError: 
 498                          # otherwise expect just these 
 499                          evname, target = em 
 500                          evmap = None 
 501                      mc.mapEvent(gdname, evname, target, evmap) 
 502          model = mc.getModel() 
 503          self._mReg[name] = model 
 504          # shortcut 
 505          self.instances = self._mReg.instances

 506   


507 -    def _infostr(self, verbose=1):


508          if verbose == 0: 
 509              outputStr = 'MProject: '+self.proj_name 
 510          elif verbose > 0: 
 511              outputStr = 'MProject: '+self.proj_name 
 512              if len(self._mReg): 
 513                  for m in self._mReg: 
 514                      outputStr += "\n" + m._infostr(verbose-1) 
 515              else: 
 516                  outputStr += 'No models in MProject '+self.proj_name 
 517          return outputStr

 518   


519 -    def __repr__(self):


520          return self._infostr(verbose=0)

 521   
 522      __str__ = __repr__ 
 523   


524 -    def info(self, verboselevel=1):


525          print self._infostr(verboselevel)

 526   
 527   
 528  # ----------------------------------------------------------------------------- 
 529   
 530  ## ModelInterface-related classes 
 531   
 532   


533 -class feature(object):


534      """End users of concrete sub-classes provide (required) evaluate method and 
 535      (optional) prepare, finish methods.""" 


536 -    def __init__(self, name, description='', pars=None, ref_traj=None):


537          self.name = name 
 538          self.description = description 
 539          if pars is None: 
 540              self.pars = common.args() 
 541          elif isinstance(pars, dict): 
 542              self.pars = common.args(**pars) 
 543          elif isinstance(pars, common.args): 
 544              self.pars = pars 
 545          else: 
 546              raise PyDSTool_TypeError("Invalid type for pars argument") 
 547          if 'verbose_level' not in self.pars: 
 548              self.pars.verbose_level = 0 
 549          if 'debug' not in self.pars: 
 550              self.pars.debug = False 
 551          # penalty used if an error occurs during residual calculation 
 552          if 'penalty' not in self.pars: 
 553              self.pars.penalty = 2000. 
 554          self.ref_traj = ref_traj 
 555          self.results = common.args() 
 556          self.super_pars = common.args() 
 557          self.super_results = common.args() 
 558          # perform any sub-class specific initializations, 
 559          # such as providing a metric (and its output array length) 
 560          try: 
 561              self._local_init() 
 562          except AttributeError: 
 563              pass 
 564          self.subfeatures = []

 565   


566 -    def __hash__(self):


567          return hash((self.name,common.className(self)))

 568   


569 -    def __eq__(self, other):


570          try: 
 571              res = self.name == other.name 
 572          except AttributeError: 
 573              return False 
 574          if hasattr(self, 'subfeatures'): 
 575              if hasattr(other, 'subfeatures'): 
 576                  res = res and self.subfeatures == other.subfeatures 
 577              else: 
 578                  return False 
 579          elif hasattr(other, 'subfeatures'): 
 580              return False 
 581          return res

 582   


583 -    def __ne__(self, other):


584          return not self == other

 585   


586 -    def _find_idx(self):


587          """Internal function for finding index in trajectory meshpoints 
 588          at which containment first failed. Defaults to returning None and 
 589          must be overridden by a class that has access to a mesh.""" 
 590          return None

 591   


592 -    def __call__(self, target):


593          try: 
 594              self.prepare(target) 
 595              satisfied = self.evaluate(target) 
 596          except KeyboardInterrupt: 
 597              raise 
 598          except: 
 599              display_error = self.pars.verbose_level > 0 or self.pars.debug 
 600              if display_error: 
 601                  exceptionType, exceptionValue, exceptionTraceback = sys.exc_info() 
 602                  print "******************************************" 
 603                  print "Problem evaluating feature:", self.name 
 604                  print "  ", exceptionType, exceptionValue 
 605                  for line in traceback.format_exc().splitlines()[-12:-1]: 
 606                      print "   " + line 
 607                  print "  originally on line:", traceback.tb_lineno(exceptionTraceback) 
 608                  if self.pars.debug:   #and self.pars.verbose_level > 1: 
 609                      raise 
 610                  else: 
 611                      print "(Proceeding as 'unsatisfied')\n" 
 612              satisfied = False 
 613              if hasattr(self, 'metric'): 
 614                  self.metric.results = self.pars.penalty * \ 
 615                                    npy.ones((self.metric_len,), float) 
 616              for sf in self.subfeatures: 
 617                  if hasattr(sf, 'metric'): 
 618                      sf.metric.results = self.pars.penalty * \ 
 619                        npy.ones((sf.metric_len,), float) 
 620          if satisfied: 
 621              self.finish(target) 
 622          self.results.satisfied = satisfied 
 623          return satisfied

 624   


625 -    def set_ref_traj(self, ref_traj):


626          """May or may not be used by the feature. If not used, 
 627          it will be ignored.""" 
 628          raise NotImplementedError("Override in concrete sub-class")

 629   


630 -    def evaluate(self, target):


631          raise NotImplementedError("Override in concrete sub-class")

 632   


633 -    def prepare(self, target):


634          """Operations to prepare for testing (optional). 
 635          Override in concrete sub-class if desired""" 
 636          pass

 637   


638 -    def finish(self, target):


639          """Operations to complete only if evaluate was True (optional). 
 640          Override in concrete sub-class if desired""" 
 641          pass

 642   


643 -    def info(self):


644          utils.info(self, self.name)

 645   


646 -    def postprocess_ref_traj(self):


647          """User-definable by overriding in a sub-class""" 
 648          pass

 649   


650 -    def validate(self):


651          """NOT YET IMPLEMENTED. Would test for reachability of a feature? 
 652          This is not easy and may be unnecessary!""" 
 653          return True

 654   


655 -    def reset_metric(self):


656          try: 
 657              self.metric.results = None 
 658          except AttributeError: 
 659              # no metric for this feature 
 660              pass

 661   
 662   
 663   


664 -class feature_leaf(feature):


665      """Abstract super-class for feature leaf nodes. 
 666      """ 


667 -    def set_ref_traj(self, ref_traj):


668          self.ref_traj = ref_traj 
 669          self.postprocess_ref_traj()

 670   


671 -    def _residual_info(self, feats, sizes):


672          """Update feats and sizes lists in place with metric info, if any. 
 673          """ 
 674          try: 
 675              sizes.append(self.metric_len) 
 676              # touch self.metric to ensure it exists! 
 677              self.metric 
 678          except AttributeError: 
 679              # no metric present 
 680              return 
 681          else: 
 682              feats.append(self)

 683   


684 -    def __str__(self):


685          return "Feature %s"%self.name

 686   
 687      __repr__ = __str__

 688   
 689   


690 -class feature_node(feature):


691      """Abstract super-class for feature regular nodes (supporting sub-features). 
 692      """ 


693 -    def __init__(self, name, description='', pars=None, 
 694                   ref_traj=None, subfeatures=None):


695          """Sub-features is an ordered sequence of QL or QT feature instances 
 696          which are (by default) evaluated in this order on a trajectory segment 
 697          unless evaluation method is overridden. 
 698   
 699          For more sophisticated use of sub-features, they should be provided as 
 700          a dictionary mapping feature names to the feature instance. 
 701          """ 
 702          feature.__init__(self, name, description, pars, ref_traj) 
 703          if subfeatures is None: 
 704              self.subfeatures = () 
 705              self._namemap = {} 
 706          elif isinstance(subfeatures, (list, tuple)): 
 707              for sf in subfeatures: 
 708                  assert isinstance(sf, feature), \ 
 709                         "Only define quantitative or qualitative features" 
 710              self.subfeatures = subfeatures 
 711              self._namemap = dict(zip([sf.name for sf in subfeatures], 
 712                                       subfeatures)) 
 713          elif isinstance(subfeatures, dict): 
 714              for sfname, sf in subfeatures.items(): 
 715                  assert isinstance(sf, feature), \ 
 716                         "Only define quantitative or qualitative features" 
 717              self.subfeatures = subfeatures 
 718              self._namemap = subfeatures 
 719          else: 
 720              raise TypeError("List or dictionary of sub-features expected")

 721   
 722   


723 -    def _residual_info(self, feats, sizes):


724          """Update feats and sizes lists in place with metric info, if any. 
 725          """ 
 726          try: 
 727              sizes.append(self.metric_len) 
 728              # touch self.metric to ensure it exists! 
 729              self.metric 
 730          except AttributeError: 
 731              # no metric present 
 732              pass 
 733          else: 
 734              feats.append(self) 
 735          # continue gathering from sub-features 
 736          for sf in self._namemap.values(): 
 737              sf._residual_info(feats, sizes)

 738   


739 -    def __str__(self):


740          s = "Feature %s "%self.name 
 741          if len(self._namemap.keys()) > 0: 
 742              s += "- " + str(self._namemap.keys()) 
 743          return s

 744   
 745      __repr__ = __str__ 
 746   


747 -    def __getitem__(self, featname):


748          """Return named sub-feature""" 
 749          return self._namemap[featname]

 750   


751 -    def propagate_verbosity(self, sf):


752          # subfeatures inherit one lower level of verbosity 
 753          if 'verbose_level' in self.pars: 
 754              v = max([0,self.pars.verbose_level - 1]) 
 755              if isinstance(sf, common._seq_types): 
 756                  for sf_i in sf: 
 757                      sf_i.pars.verbose_level = v 
 758              else: 
 759                  sf.pars.verbose_level = v 
 760          if 'debug' in self.pars: 
 761              if isinstance(sf, common._seq_types): 
 762                  for sf_i in sf: 
 763                      sf_i.pars.debug = self.pars.debug 
 764              else: 
 765                  sf.pars.debug = self.pars.debug

 766   


767 -    def evaluate(self, target):


768          """Default method: evaluate sub-features in order (assumes they 
 769          are stored as a list). 
 770   
 771          Can override with more sophisticated method (e.g. for use with a 
 772          dictionary of sub-features). 
 773          """ 
 774          # initial value 
 775          satisfied = True 
 776          # this loop only works if subfeatures is a list 
 777          # (must retain correct order for this list so don't use _namemap.values()) 
 778          for sf in self.subfeatures: 
 779              try: 
 780                  self.propagate_verbosity(sf) 
 781              except KeyboardInterrupt: 
 782                  raise 
 783              except: 
 784                  if not isinstance(self.subfeatures, common._seq_types): 
 785                      raise TypeError("You must override the evaluate method for " 
 786                                      "dictionary-based sub-features") 
 787                  else: 
 788                      raise 
 789              sf.super_pars.update(self.pars) 
 790              sf.super_results.update(self.results) 
 791              sf.reset_metric() 
 792              if self.pars.verbose_level > 1: 
 793                  print "feature_node.evaluate: sf=", sf 
 794              error_raised = False 
 795              try: 
 796                  new_result = sf(target) 
 797              except KeyboardInterrupt: 
 798                  raise 
 799              except: 
 800                  # catch errors in prepare or finish (evaluate was trapped 
 801                  # in __call__) 
 802                  new_result = False 
 803                  error_raised = True 
 804                  if sf.pars.debug: # and sf.pars.verbose_level > 1: 
 805                      raise 
 806              # have to compute new separately to ensure sf computes its results 
 807              # for potential use by a residual function 
 808              satisfied = satisfied and new_result 
 809              if error_raised: 
 810                  print " ... error raised" 
 811                  if hasattr(self, 'metric'): 
 812                      # kludgy penalty function in lieu of something smarter 
 813                      if sf.metric.results is None: 
 814                          sf.metric.results = self.pars.penalty * \ 
 815                            npy.ones((sf.metric_len,),float) 
 816              else: 
 817                  self.results.update(sf.results) 
 818          return satisfied

 819   


820 -    def set_ref_traj(self, ref_traj):


821          """May or may not be used by the feature. If not used, it will be 
 822          ignored.""" 
 823          self.ref_traj = ref_traj 
 824          self.postprocess_ref_traj() 
 825          if isinstance(self.subfeatures, dict): 
 826              sfs = self.subfeatures.values() 
 827          else: 
 828              sfs = self.subfeatures 
 829          for sf in sfs: 
 830              self.propagate_verbosity(sf) 
 831              sf.super_pars.update(self.pars) 
 832              sf.super_results.update(self.results) 
 833              sf.set_ref_traj(ref_traj) 
 834              self.pars.update(sf.pars)

 835   
 836   


837 -class ql_feature_leaf(feature_leaf):


838      """Qualitative feature (leaf node). 
 839      Add description to note assumptions used for defining feature. 
 840   
 841      input: a trajectory segment 
 842      output: a vector of boolean-valued event detections (non-terminal events or even 
 843        non-linked python events) or other function tests (e.g. existence of a fixed point) 
 844        stored in a list. 
 845      """

 846   
 847   


848 -class qt_feature_leaf(feature_leaf):


849      """Quantitative feature (leaf node). 
 850      Add description to note assumptions used for defining feature. 
 851   
 852      input: a trajectory segment 
 853      output: a vector of boolean-valued tolerance tests on the discrepancies between ideal and 
 854        actual features defined by a list of function tests 
 855        e.g. a test returns (residual of ideal-actual) < tolerance 
 856      """

 857   
 858   


859 -class ql_feature_node(feature_node):


860      """Qualitative feature (regular node). 
 861      Add description to note assumptions used for defining feature. 
 862   
 863      input: a trajectory segment 
 864      output: a vector of boolean-valued event detections (non-terminal events or even 
 865        non-linked python events) or other function tests (e.g. existence of a fixed point) 
 866        stored in a list. 
 867      """

 868   
 869   


870 -class qt_feature_node(feature_node):


871      """Quantitative feature (regular node). 
 872      Add description to note assumptions used for defining feature. 
 873   
 874      input: a trajectory segment 
 875      output: a vector of boolean-valued tolerance tests on the discrepancies between ideal and 
 876        actual features defined by a list of function tests 
 877        e.g. a test returns (residual of ideal-actual) < tolerance 
 878      """

 879   
 880  # ----------------------------------------------------------------------------- 
 881   
 882   


883 -class condition(object):


884      """Model context condition, made up of a boolean composition of wanted and 
 885      unwanted features. 
 886      This is specified by a dictionary of feature objects mapping to True 
 887      (wanted feature) or False (unwanted feature). 
 888      """ 
 889   


890 -    def __init__(self, feature_composition_dict):


891          # fcd maps feature objects to True (wanted feature) or 
 892          # False (unwanted feature) 
 893          self.namemap = {} 
 894          try: 
 895              for f, c in feature_composition_dict.iteritems(): 
 896                  assert isinstance(c, bool), \ 
 897                         "Feature composition dictionary requires boolean values" 
 898                  assert isinstance(f, (ql_feature_leaf, qt_feature_leaf, 
 899                                        ql_feature_node, qt_feature_node)), \ 
 900                          "Only define quantitative or qualitative features" 
 901                  self.namemap[f.name] = f 
 902          except AttributeError: 
 903              raise TypeError("Dictionary of features to Booleans expected") 
 904          self.fcd = feature_composition_dict 
 905          self.results = common.args()

 906   


907 -    def __eq__(self, other):


908          try: 
 909              return self.namemap == other.namemap 
 910          except AttributeError: 
 911              return False

 912   


913 -    def __ne__(self, other):


914          try: 
 915              return self.namemap != other.namemap 
 916          except AttributeError: 
 917              return True

 918   


919 -    def keys(self):


920          return self.namemap.keys()

 921   


922 -    def values(self):


923          return self.namemap.values()

 924   


925 -    def items(self):


926          return self.namemap.items()

 927   


928 -    def __getitem__(self, name):


929          return self.namemap[name]

 930   


931 -    def __contains__(self, name):


932          return name in self.namemap

 933   


934 -    def set_ref_traj(self, ref_traj):


935          """Set reference trajectory for the features (if used, otherwise will 
 936          be ignored or overridden in feature _local_init methods). 
 937          """ 
 938          for f,c in self.fcd.iteritems(): 
 939              f.set_ref_traj(ref_traj)

 940   


941 -    def evaluate(self, target):


942          """Apply conditions to trajectory segments 
 943          and returns True if all are satisfied.""" 
 944          satisfied = True 
 945          for f,c in self.fcd.iteritems(): 
 946              # have to call new separately to ensure f calcs its residual 
 947              new = f(target) == c 
 948              satisfied = satisfied and new 
 949              self.results[f.name] = f.results 
 950          return satisfied

 951   
 952      __call__ = evaluate 
 953   


954 -    def __str__(self):


955          s = "Condition " 
 956          if len(self.namemap.keys()) > 0: 
 957              s += "- " + str(self.namemap.keys()) 
 958          return s

 959   
 960      __repr__ = __str__ 
 961   


962 -    def _find_idx(self):


963          min_ix = npy.Inf 
 964          for f in self.fcd.keys(): 
 965              f_ix = f._find_idx() 
 966              if f_ix is not None and f_ix < min_ix: 
 967                  min_ix = f_ix 
 968          if npy.isfinite(min_ix): 
 969              return min_ix 
 970          else: 
 971              return None

 972   


973 -    def _residual_info(self):


974          """Update metric information used for residual / objective function, 
 975          from all sub-features.""" 
 976          #feats and sizes updated in place 
 977          feats = [] 
 978          sizes = [] 
 979          for f in self.fcd.keys(): 
 980              f._residual_info(feats, sizes) 
 981          return {'features': dict(zip(feats,sizes)), 
 982                  'total_size': sum(sizes)}

 983   


984 -    def collate_results(self, result_name, merge_lists=False, 
 985                          feature_names=None):


986          res = [] 
 987          if feature_names is None: 
 988              feature_list = self.fcd.keys() 
 989          else: 
 990              feature_list = [self.namemap[f] for f in feature_names] 
 991          for f in feature_list: 
 992              try: 
 993                  resval = getattr(f.results, result_name) 
 994              except AttributeError: 
 995                  # no such result name 
 996                  continue 
 997              else: 
 998                  if merge_lists and isinstance(resval, list): 
 999                      res.extend(resval) 
1000                  else: 
1001                      res.append(resval) 
1002          return res

1003   
1004   


1005 -class context(object):


1006      """A collection of related model interfaces that apply to a model. 
1007      interface_pairs are a list of ModelInterface instance (test) and class (ref) pairs, 
1008      the latter to be instantiated on a model. 
1009   
1010      Set the debug_mode attribute at any time, or as the optional argument at initializiation, 
1011      to ensure that any exceptions that arise from interacting model interfaces and their 
1012      features are fully passed back to the caller of the context containing them. 
1013      """ 


1014 -    def __init__(self, interface_pairs, debug_mode=False):


1015          self.interfaces = dict(interface_pairs) 
1016          self.debug_mode = debug_mode 
1017          # Determine which qt features have metrics to use to make a 
1018          # residual function. Keep multiple views of this data for efficient 
1019          # access in different ways. 
1020          metric_features = {} 
1021          res_feature_list = [] 
1022          tot_size = 0 
1023          for test_mi, ref_mi_class in self.interfaces.iteritems(): 
1024              # list of suitable features for each test_mi 
1025              metric_features[test_mi] = test_mi.conditions._residual_info() 
1026              tot_size += metric_features[test_mi]['total_size'] 
1027              res_feature_list.extend([(test_mi, f) for f in \ 
1028                                       metric_features[test_mi]['features'].keys()]) 
1029          self.metric_features = metric_features 
1030          self.res_feature_list = res_feature_list 
1031          self.res_len = tot_size 
1032          # instances cleared on each evaluation of a model 
1033          self.ref_interface_instances = [] 
1034          # default weights are all 1, and set up weights dictionary 
1035          self.reset_weights()

1036   


1037 -    def reset_weights(self, old_weights=None):


1038          """Reset weights to unity, unless old_weights array 
1039          is given, in which case reset to that. 
1040          """ 
1041          if old_weights is None: 
1042              self.weights = npy.ones(self.res_len, 'float') 
1043          else: 
1044              self.weights = old_weights 
1045          self.weight_index_mapping = {} 
1046          self.feat_weights = {} 
1047          ix = 0 
1048          for test_mi, feat_dict in self.metric_features.iteritems(): 
1049              self.weight_index_mapping[test_mi] = {} 
1050              for f, size in feat_dict['features'].iteritems(): 
1051                  self.weight_index_mapping[test_mi][f] = (ix, ix+size) 
1052                  # weights are constant for a given feature 
1053                  self.feat_weights[(test_mi, f)] = self.weights[ix] 
1054                  ix += size

1055   


1056 -    def set_single_feat_weights(self, feat, weight=1):


1057          """Set weights for a single feature given as an (interface, feature) 
1058          pair, setting all others to zero.""" 
1059          wdict = {} 
1060          for test_mi, feat_dict in self.metric_features.iteritems(): 
1061              if test_mi != feat[0]: 
1062                  continue 
1063              w = {}.fromkeys(feat_dict['features'].keys(), 0) 
1064              if feat[1] in w: 
1065                  w[feat[1]] = weight 
1066              wdict[test_mi] = w 
1067          self.set_weights(wdict)

1068   


1069 -    def set_weights(self, weight_dict):


1070          """Update weights with a dictionary keyed by test_mi, whose values are 
1071          either: 
1072           (1) dicts of feature -> scalar weight. 
1073           (2) a scalar which will apply to all features of that model interface 
1074          Features and model interfaces must correspond to those declared for the 
1075          context. 
1076          """ 
1077          for test_mi, fs in weight_dict.iteritems(): 
1078              try: 
1079                  flist = self.metric_features[test_mi]['features'].keys() 
1080              except KeyError: 
1081                  raise AssertionError("Invalid test model interface") 
1082              if isinstance(fs, common._num_types): 
1083                  feat_dict = {}.fromkeys(flist, fs) 
1084              elif isinstance(fs, dict): 
1085                  assert npy.alltrue([isinstance(w, common._num_types) for \ 
1086                              w in fs.values()]), "Invalid scalar weight" 
1087                  assert npy.alltrue([f in flist for f in fs.keys()]), \ 
1088                         "Invalid features given for this test model interface" 
1089                  feat_dict = fs 
1090              for f, w in feat_dict.iteritems(): 
1091                  self.feat_weights[(test_mi, f)] = w 
1092                  # update weight value 
1093                  start_ix, end_ix = self.weight_index_mapping[test_mi][f] 
1094                  self.weights[start_ix:end_ix] = w

1095   


1096 -    def show_res_info(self, resvec):


1097          """Show detail of feature -> residual mapping for a given residual 
1098          vector.""" 
1099          i = 0 
1100          for test_mi, feat_dict in self.metric_features.iteritems(): 
1101              print "Test model interface:", test_mi 
1102              for f in feat_dict['features']: 
1103                  if self.feat_weights[(test_mi, f)] == 0: 
1104                      continue 
1105                  ix0, ix1 = self.weight_index_mapping[test_mi][f] 
1106                  len_w = ix1-ix0 
1107                  f_str = "  "+f.name 
1108                  # '  unweighted:' is 13 chars long 
1109                  extra_space_w = " "*max([0, 13-len(f_str)]) 
1110                  extra_space_unw = " "*max([0, len(f_str)-13]) 
1111                  print f_str + extra_space_w , resvec[i:i+len_w] 
1112                  try: 
1113                      print "  unweighted:" + extra_space_unw, \ 
1114                             resvec[i:i+len_w]/self.weights[ix0:ix1] 
1115                  except ZeroDivisionError: 
1116                      print "  (unweighted values unavailable)" 
1117                  i += len_w

1118   


1119 -    def _map_to_features(self, x):


1120          """Utility to map 1D array x onto the model interface's 
1121          features with non-zero weights, returning a dictionary. 
1122   
1123          x is assumed to have correct length. 
1124          """ 
1125          out = {} 
1126          i = 0 
1127          for test_mi, feat_dict in self.metric_features.iteritems(): 
1128              for f in feat_dict['features']: 
1129                  if self.feat_weights[(test_mi, f)] == 0: 
1130                      continue 
1131                  ix0, ix1 = self.weight_index_mapping[test_mi][f] 
1132                  len_w = ix1-ix0 
1133                  try: 
1134                      out[test_mi][f] = x[i:i+len_w] 
1135                  except KeyError: 
1136                      out[test_mi] = {f: x[i:i+len_w]} 
1137                  i += len_w 
1138          return out

1139   


1140 -    def evaluate(self, model):


1141          """Evaluate whole context on a model instance, returning a single 
1142          Boolean. 
1143          """ 
1144          result = True 
1145          # typically, test_mi is an external interface (e.g., for data) 
1146          # and ref_mi is an internal interface (e.g., for a model) 
1147          self.ref_interface_instances = [] 
1148          for test_mi, ref_mi_class in self.interfaces.iteritems(): 
1149              # evaluate test_mi on model, via the associated ref_mi 
1150              ref_mi = ref_mi_class(model) 
1151              self.ref_interface_instances.append(ref_mi) 
1152              try: 
1153                  new_result = test_mi(ref_mi) 
1154              except KeyboardInterrupt: 
1155                  raise 
1156              except: 
1157                  if self.debug_mode: 
1158                      raise 
1159                  else: 
1160                      print "******************************************" 
1161                      print "Problem evaluating interface", test_mi, "on ", ref_mi 
1162                      print "  ", sys.exc_info()[0], sys.exc_info()[1] 
1163                      new_result = False 
1164              # must create new_res first, to ensure all interfaces are 
1165              # evaluated (to create their results for possible post-processing) 
1166              result = result and new_result 
1167          return result

1168   


1169 -    def residual(self, model, include_raw=False):


1170          """Evaluate whole context on a model instance, returning an array 
1171          of residual error between quantitative features in the model trajectory 
1172          and their target values. 
1173   
1174          Residual array will be weighted if one was set. Any weights set to zero 
1175          will cause those features to *not appear* in the residual. 
1176   
1177          Provide include_raw=True argument to also return the raw, unweighted residual. 
1178          (Mainly for internal use.) 
1179          """ 
1180          # discard the boolean, just compute the residuals through the calls to 
1181          # metric, and access them through the feature list 
1182          self.evaluate(model) 
1183          raw_residual = npy.concatenate(tuple([mf[1].metric.results for \ 
1184                                       mf in self.res_feature_list])) 
1185          residual = process_raw_residual(raw_residual, self.weights) 
1186          if include_raw: 
1187              return residual, raw_residual 
1188          else: 
1189              return residual

1190   
1191   


1192 -def process_raw_residual(raw_residual, weights):


1193      ixs = npy.nonzero(weights) 
1194      residual = (weights*raw_residual)[ixs] 
1195      nan_ixs = npy.where(npy.asarray(npy.isnan(residual),int)) 
1196      for ix in nan_ixs: 
1197          residual[ix] = 100. 
1198      return residual

1199   
1200   
1201  # ----------------------------------------------------------------- 
1202   


1203 -class always_feature(ql_feature_leaf):


1204      """Use this for a single vector field model that uses discrete 
1205      event mappings.""" 


1206 -    def evaluate(self, target):


1207          return True

1208   


1209 -class binary_feature(ql_feature_leaf):


1210      """Use this as a binary switch feature, toggled 
1211      by a given variable name 'varname' that is supplied 
1212      in the pars dict at initialization.""" 


1213 -    def evaluate(self, target):


1214          try: 
1215              pts = target.test_traj.sample(coords=[self.pars.varname]) 
1216          except AttributeError: 
1217              raise AttributeError("No variable name given for switch") 
1218          except KeyboardInterrupt: 
1219              raise 
1220          except: 
1221              print "Failed to find trajectory values for given variable name: %s"%self.pars.varname 
1222              raise 
1223          self.results.output = pts 
1224          return all(self.results.output==1)

1225   


1226 -    def _find_idx(self):


1227          if self.results.satisfied: 
1228              # Trajectory satisfied contraint! 
1229              return None 
1230          res = self.results.output 
1231          if res[0] == 1: 
1232              adjusted_res = list((res - 1) != 0) 
1233          else: 
1234              if 1 not in res: 
1235                  # never goes to excited state so no index to return 
1236                  raise RuntimeError 
1237              adjusted_res = list(res != 0) 
1238          # find first index at which value is non-zero 
1239          # should never raise ValueError because this method is 
1240          # only run if there was a sign change found 
1241          return adjusted_res.index(True)

1242   
1243   
1244  # ---------------------------------------------------------------------------- 
1245   


1246 -class dsInterface(object):


1247      """Generic and abstract interface class for dynamical systems.""" 
1248  #    _getkeys = ['indepvariable', 'algparams', 'funcspec', 
1249  #                   'diagnostics', 'variables', 
1250  #                   'pars', 'inputs', 
1251  #                   'eventstruct', 'globalt0', 'Rhs', 'Jacobian', 
1252  #                   'JacobianP', 'MassMatrix', 'AuxVars'] 
1253      _setkeys = ['globalt0', 'tdata', 'pars', 'algparams', 'inputs'] 
1254      # the query key list is copied from Model.Model 
1255      _querykeys = ['pars', 'parameters', 'events', 'submodels', 
1256                    'ics', 'initialconditions', 'vars', 'variables', 
1257                    'auxvariables', 'auxvars',  'vardomains', 'abseps'] 
1258   


1259 -    def get_test_traj(self):


1260          raise NotImplementedError("Only call this on a concrete sub-class")

1261   


1262 -    def query(self, querykey=''):


1263          return self.model.query(querykey)

1264   
1265   


1266 -class GeneratorInterface(dsInterface):


1267      """Wrapper for Generator (for non-hybrid models) that shares similar API 
1268      with ModelInterface for use in HybridModel objects.""" 
1269   


1270 -    def __init__(self, model, FScompatibleNames=None, 
1271                   FScompatibleNamesInv=None):


1272          """model argument must be a Generator only""" 
1273          self.model = model 
1274          if FScompatibleNames is None: 
1275              if self.model._FScompatibleNames is None: 
1276                  self.model._FScompatibleNames = symbolMapClass() 
1277          else: 
1278              self.model._FScompatibleNames = FScompatibleNames 
1279          if FScompatibleNamesInv is None: 
1280              if self.model._FScompatibleNamesInv is None: 
1281                  self.model._FScompatibleNamesInv = symbolMapClass() 
1282          else: 
1283              self.model._FScompatibleNamesInv = FScompatibleNamesInv 
1284          self.eventstruct = Events.EventStruct()

1285          #self.diagnostics = common.Diagnostics() 
1286   


1287 -    def get(self, key, ics=None, t0=0):


1288          # self.model is a Generator 
1289          return self.model.get(key)

1290   


1291 -    def set(self, key, value, ics=None, t0=0):


1292          if key in self._setkeys: 
1293              self.model.set(**{key:value}) 
1294          else: 
1295              raise KeyError("Invalid or unsupported 'set' key: %s"%key)

1296   


1297 -    def Rhs(self, t, xdict, pdict):


1298          """Direct access to a generator's Rhs function.""" 
1299          return self.model.Rhs(t, xdict, pdict)

1300   


1301 -    def Jacobian(self, t, xdict, pdict, idict=None):


1302          """Direct access to a generator's Jacobian function (if defined).""" 
1303          return self.model.Jacobian(t, xdict, pdict)

1304   


1305 -    def JacobianP(self, t, xdict, pdict):


1306          """Direct access to a generator's JacobianP function (if defined).""" 
1307          return self.model.JacobianP(t, xdict, pdict)

1308   


1309 -    def MassMatrix(self, t, xdict, pdict):


1310          """Direct access to a generator's MassMatrix function (if defined).""" 
1311          return self.model.MassMatrix(t, xdict, pdict)

1312   


1313 -    def AuxVars(self, t, xdict, pdict):


1314          """Direct access to a generator's auxiliary variables 
1315          definition (if defined).""" 
1316          return self.model.AuxVars(t, xdict, pdict)

1317   
1318   


1319 -class ModelInterface(dsInterface):


1320      """Model constraints expressed as a uni-directional interface to another 
1321      formal system model: 
1322      - Made up of conditions imposed on the other system's test trajectory. 
1323      - Defines evaluation criteria for any view (e.g. from experimental data and 
1324      test conditions). 
1325      This is an abstract superclass for the 'internal' and 'external' 
1326      sub-classes. 
1327      """ 
1328      _trajname = 'test_iface_traj' 
1329   


1330 -    def __init__(self):


1331          # Cache (3-tuple) for the ics, t0 and initiator last specified 
1332          self._initiator_cache = None 
1333          self.eventstruct = Events.EventStruct()

1334          #self.diagnostics = common.Diagnostics() 
1335   


1336 -    def _get_initiator_cache(self, ics=None, t0=0):


1337          """initiator is a ModelInterface or GeneratorInterface object""" 
1338          if self._initiator_cache is None: 
1339              if ics is None: 
1340                  raise ValueError("Must pass initial conditions") 
1341              else: 
1342                  initiator = self.model._findTrajInitiator(None, 0, 
1343                                                  t0, dict(ics))[0] 
1344                  self._initiator_cache = (ics, t0, initiator) 
1345          else: 
1346              if npy.alltrue(self._initiator_cache[0] == ics) and \ 
1347                 self._initiator_cache[1] == t0: 
1348                  ics, t0, initiator = self._initiator_cache 
1349              elif ics is None: 
1350                  raise ValueError("Must pass initial conditions") 
1351              else: 
1352                  # initial conditions or t0 don't match -- don't use cache 
1353                  initiator = self.model._findTrajInitiator(None, 0, 
1354                                                  t0, dict(ics))[0] 
1355                  self._initiator_cache = (ics, t0, initiator) 
1356          return (ics, t0, initiator)

1357   


1358 -    def set(self, key, value, ics=None, t0=0):


1359          # ModelInterface.set: something is not good about this structure! 
1360          self.model.set(**{key:value}) 
1361          ics, t0, initiator = self._get_initiator_cache(ics, t0) 
1362  #        print "ModelInterface.set %s = %s for %s (type %s)"%(str(key), str(value), initiator.model.name, type(initiator)) 
1363          if key in self._setkeys: 
1364              initiator.set(key, value, ics, t0) 
1365              initiator.model.set(**{key:value}) 
1366          else: 
1367              raise KeyError("Invalid or unsupported 'set' key: %s"%key)

1368   


1369 -    def get(self, key, ics=None, t0=0):


1370          ics, t0, initiator = self._get_initiator_cache(ics, t0) 
1371  #        if key in self._getkeys: 
1372          try: 
1373              return initiator.get(key, ics, t0) 
1374          except AttributeError: 
1375              raise ValueError("Invalid or unsupported 'get' key: %s"%key)

1376   


1377 -    def Rhs(self, t, xdict, pdict):


1378          """Direct access to a generator's Rhs function.""" 
1379          ics_ignore, t_ignore, ds = self._get_initiator_cache(xdict, t) 
1380          try: 
1381              return self.model.Rhs(ds._supermodel.name, t, xdict, pdict) 
1382          except AttributeError: 
1383              # ds is not a MI with attribute _supermodel 
1384              return self.model.Rhs(t, xdict, pdict)

1385   


1386 -    def Jacobian(self, t, xdict, pdict, idict=None):


1387          """Direct access to a generator's Jacobian function (if defined).""" 
1388          ics_ignore, t_ignore, ds = self._get_initiator_cache(xdict, t) 
1389          try: 
1390              return self.model.Jacobian(ds._supermodel.name, t, xdict, pdict) 
1391          except AttributeError: 
1392              # ds is not a MI with attribute _supermodel 
1393              return self.model.Jacobian(t, xdict, pdict)

1394   


1395 -    def JacobianP(self, t, xdict, pdict):


1396          """Direct access to a generator's JacobianP function (if defined).""" 
1397          ics_ignore, t_ignore, ds = self._get_initiator_cache(xdict, t) 
1398          try: 
1399              return self.model.JacobianP(ds._supermodel.name, t, xdict, pdict) 
1400          except AttributeError: 
1401              # ds is not a MI with attribute _supermodel 
1402              return self.model.JacobianP(t, xdict, pdict)

1403   


1404 -    def MassMatrix(self, t, xdict, pdict):


1405          """Direct access to a generator's MassMatrix function (if defined).""" 
1406          ics_ignore, t_ignore, ds = self._get_initiator_cache(xdict, t) 
1407          try: 
1408              return self.model.MassMatrix(ds._supermodel.name, t, xdict, pdict) 
1409          except AttributeError: 
1410              # ds is not a MI with attribute _supermodel 
1411              return self.model.MassMatrix(t, xdict, pdict)

1412   


1413 -    def AuxVars(self, t, xdict, pdict):


1414          """Direct access to a generator's auxiliary variables 
1415          definition (if defined).""" 
1416          ics_ignore, t_ignore, ds = self._get_initiator_cache(xdict, t) 
1417          try: 
1418              return ds.model.AuxVars(ds._supermodel.name, t, xdict, pdict) 
1419          except AttributeError: 
1420              # ds is not a MI with attribute _supermodel 
1421              return ds.model.AuxVars(t, xdict, pdict)

1422   


1423 -    def setup_conditions(self, conditions, traj):


1424          # in case the conditions use this model trajectory as a reference 
1425          # then provide them with it 
1426          if conditions is None: 
1427              self.conditions = None 
1428          else: 
1429              # really need to copy conditions? 
1430              self.conditions = conditions 
1431              try: 
1432                  self.conditions.set_ref_traj(traj) 
1433              except AttributeError: 
1434                  raise

1435   


1436 -    def evaluate(self, target, force=False):


1437          """Evaluate interface consistency against target interface's trajectory 
1438          on specified conditions. 
1439   
1440          Optional force argument forces model to recompute its test trajectory, 
1441          e.g. because of a known change in model parameters, ics, etc. 
1442          """ 
1443          assert isinstance(target, ModelInterface), \ 
1444                 "Target argument must be another interface object" 
1445          if len(self.compatibleInterfaces) > 0 and \ 
1446               target.__class__.__name__ not in self.compatibleInterfaces \ 
1447                  and not npy.sometrue([common.compareBaseClass(target, ctype) \ 
1448                                       for ctype in self.compatibleInterfaces]): 
1449              raise ValueError("Target interface not of compatible type") 
1450          try: 
1451              self.conditions 
1452          except AttributeError: 
1453              self.setup_conditions(conditions, self.get_test_traj()) 
1454          force = force or target.test_traj is None 
1455          if force: 
1456              # discard returned traj here (still accessible via target.test_traj) 
1457              target.get_test_traj(force=force) 
1458          self.prepare_conditions(target) 
1459          try: 
1460              result = self.conditions(target) 
1461          except KeyError: 
1462              raise KeyError("Condition evaluation failed") 
1463          return result

1464   
1465      __call__ = evaluate 
1466   


1467 -    def postprocess_test_traj(self, test_traj):


1468          """Called by another interface via get_test_traj. 
1469          Default post-processing of test trajectory is the identity 
1470           function, i.e. no processing. 
1471   
1472          Override this method to return a processed version of the 
1473           trajectory or perform other post-computation clean-up, e.g. 
1474           prepare auxiliary feature/condition-related information based 
1475           on end state of trajectory so that HybridModel can use it to 
1476           decide on next hybrid state to switch to. 
1477          """ 
1478          return test_traj

1479   


1480 -    def prepare_conditions(self, target):


1481          """Called automatically by evaluate. Override with user-defined access 
1482          to the target interface or processing of trajectory after return of the 
1483          target's test trajectory. 
1484          """ 
1485          pass

1486   
1487   


1488 -class intModelInterface(ModelInterface):


1489      """Interface providing internal evaluation criteria between models. 
1490      Optional conditions (object) argument used to specify these criteria. 
1491      """ 


1492 -    def __init__(self, model, conditions=None, compatibleInterfaces=None, 
1493                   test_traj=None):


1494          """Set model that generates test trajectories from which the dictionary 
1495          of conditions can be imposed on a connected model. 
1496   
1497          If no conditions are specified then the model is trivially wrapped in 
1498            an "empty" interface. 
1499   
1500          Optionally, a dummy test traj can be supplied in case of a dummy interface 
1501          for a trivial condition test that does not need to evaluate a trajectory 
1502          to determine the result. 
1503          """ 
1504          ModelInterface.__init__(self) 
1505          assert isinstance(model, Model.Model), "Invalid Model object passed" 
1506          self.model = model  #copy.deepcopy(model)  # ??? 
1507          #print "TEMP: (intModelInterface.__init__) -- should model be copied?" 
1508          self.test_traj = test_traj  # may be initially a temporary value, None

1509   


1510 -    def ensure_has_test_traj(self):


1511          """Cause recomputation of test trajectory if not already present in 
1512          model, returning boolean for whether recomputation was performed. 
1513          """ 
1514          info = self.model.current_defining_args() 
1515          # include any interface-specific changes that would be made 
1516          new_args = self.initialize_model() 
1517          if new_args is not None: 
1518              info.update(new_args) 
1519          if self.model.has_exact_traj(self._trajname, info): 
1520              # this verifies that the traj that would be computed 
1521              # already exists 
1522              return False 
1523          else: 
1524              try: 
1525                  self.compute_traj(need_init=False, new_args=new_args) 
1526              except KeyboardInterrupt: 
1527                  raise 
1528              except: 
1529                  print "Model interface compute_traj method for model " + \ 
1530                        "'%s' failed" % self.model.name 
1531                  print sys.exc_info()[0], sys.exc_info()[1] 
1532                  return False 
1533              else: 
1534                  return True

1535   


1536 -    def has_test_traj(self):


1537          return self.test_traj is not None

1538   


1539 -    def compute_traj(self, need_init=True, new_args=None):


1540          if need_init: 
1541              new_args = self.initialize_model() 
1542          if new_args is not None and len(new_args) > 0: 
1543              old_info = self.model.current_defining_args() 
1544              self.model.set(**new_args) 
1545          else: 
1546              old_info = None 
1547          self.model.compute(trajname=self._trajname, force=True) 
1548          if old_info is not None: 
1549              # restore "standard" state 
1550              self.model.set(**dict(old_info))

1551   


1552 -    def get_test_traj(self, force=False):


1553          """Called by another interface. 
1554          Return model's test trajectory, using any post-processing 
1555          specified by user-defined process_test_traj method. 
1556   
1557          Use force option if model is known to have changed and trajectory 
1558          needs refreshing. 
1559          """ 
1560          if force and not isinstance(self.test_traj, Trajectory.Trajectory): 
1561              self.compute_traj() 
1562              recomputed = True 
1563          else: 
1564              recomputed = self.ensure_has_test_traj() 
1565          if recomputed or self.test_traj is None: 
1566              self.test_traj = \ 
1567                  self.postprocess_test_traj(self.model[self._trajname]) 
1568          return self.test_traj

1569   


1570 -    def initialize_model(self):


1571          """Return any unique model-specific settings here, as a dictionary with 
1572          keys that can include initial conditions, parameters, tdata, algorithmic 
1573          parameters. Use the same keys that are suitable for a call to the 
1574          Model.set method, i.e. 'pars', 'ics', 'tdata', and 'algparams'. 
1575   
1576          Override in a sub-class to use. This method will be called 
1577          before any trajectory computation of the model. 
1578          """ 
1579          pass

1580   
1581   


1582 -class extModelInterface(ModelInterface):


1583      """Interface from a trajectory of numerical data and test conditions 
1584      providing external evaluation criteria for a model. 
1585      Optional conditions (object) argument used to specify these criteria. 
1586      """ 


1587 -    def __init__(self, traj=None, conditions=None, compatibleInterfaces=None):


1588          ModelInterface.__init__(self) 
1589          self.setup_conditions(conditions, traj) 
1590          self.set_test_traj(traj) 
1591          if compatibleInterfaces is None: 
1592              self.compatibleInterfaces = [] 
1593          else: 
1594              self.compatibleInterfaces = compatibleInterfaces

1595   


1596 -    def set_test_traj(self, traj):


1597          """Do any user-defined preprocessing to the given trajectory, including 
1598          converting it to a different type of trajectory. 
1599          """ 
1600          self.test_traj = self.postprocess_test_traj(traj) 
1601          # propagate ref traj to conditions if they use it 
1602          self.conditions.set_ref_traj(self.test_traj)

1603   


1604 -    def ensure_has_test_traj(self):


1605          """Never needs to recompute trajectory as it is fixed, so always 
1606          returns False. 
1607          """ 
1608          assert self.has_test_traj(), "Test trajectory missing" 
1609          return False

1610   


1611 -    def has_test_traj(self):


1612          return isinstance(self.test_traj, Trajectory.Trajectory)

1613   


1614 -    def get_test_traj(self, force=False):


1615          """Called by another interface. 
1616          Optional force argument is ignored for this class, as the 
1617           trajectory is fixed.""" 
1618          return self.test_traj

1619   
1620   
1621   
1622  # old code for internal interface 
1623              ## currently both 'ics' and 'initialconditions' keys are valid, but want only one 
1624              ## present here and we'll make sure it's stored as 'ics' here 
1625              #assert len(common.intersect(["initialconditions", "ics"], conditions.keys())) == 1, \ 
1626                     #"Conditions must include one list of initial conditions" 
1627              #if 'ics' not in conditions: 
1628                  #self.conditions['ics'] = conditions['initialconditions'] 
1629                  #del self.conditions['initialconditions'] 
1630   
1631  # ---------------------------------------------------------------------------- 
1632   
1633  # Private classes 
1634   


1635 -class MReg(object):


1636      """Registry class for Model descriptors and instances in Model projects. 
1637      For internal use by PyDSTool.""" 
1638   


1639 -    def __init__(self):


1640          # for model descriptors 
1641          self.descs = {} 
1642          # for associated model instances 
1643          self.instances = {}

1644   


1645 -    def add(self, descriptor):


1646          """descriptor expected to be an MDescriptor object. 
1647          """ 
1648          if isinstance(descriptor, ModelConstructor.MDescriptor): 
1649              self.descs[descriptor.name] = descriptor 
1650              self.instances[descriptor.name] = {} 
1651          else: 
1652              raise TypeError("Only MDescriptor objects valid for MReg class")

1653   


1654 -    def __setitem__(self, name, model_instance):


1655          try: 
1656              self.instances[name] = model_instance 
1657          except KeyError: 
1658              raise ValueError("No such model descriptor")

1659   


1660 -    def __contains__(self, name):


1661          return name in self.descs

1662   


1663 -    def __getitem__(self, name):


1664          return self.descs[name]

1665   


1666 -    def __delitem__(self, name):


1667          del(self.descs[name]) 
1668          del(self.instances[name])

1669   
1670      remove = __delitem__ 
1671   


1672 -    def query(self, querykey, value):


1673          """Return info about stored model specifications. 
1674          Valid query keys: 'orig_name', 'in_description' 
1675          """ 
1676          assert isinstance(querykey, str), \ 
1677                         ("Query argument must be a single string") 
1678          _keylist = ['orig_name', 'in_description'] 
1679          if querykey not in _keylist: 
1680              print 'Valid query keys are:', _keylist 
1681              raise TypeError('Query key '+querykey+' is not valid') 
1682          if querykey == 'orig_name': 
1683              res = [] 
1684              for name, regentry in self.descs.iteritems(): 
1685                  if regentry.orig_name == value: 
1686                      res.append(name) 
1687              return res 
1688          if querykey == 'in_description': 
1689              res = [] 
1690              for name, regentry in self.descs.iteritems(): 
1691                  if value in regentry.description: 
1692                      res.append(name) 
1693              return res

1694
```

  


| Home | Trees | Indices | Help | PyDSTool |
| --- | --- | --- | --- | --- |

|  |  |
| --- | --- |
| Generated by Epydoc 3.0.1 on Fri May 4 15:24:18 2012 | http://epydoc.sourceforge.net |
